# Disparities in Cardiovascular Health by Food Security Status and Supplemental Nutrition Assistance Program Participation Using Life’s Essential 8 Metrics

**DOI:** 10.1001/jamanetworkopen.2023.21375

**Published:** 2023-06-30

**Authors:** Cindy W. Leung, Julia A. Wolfson, Eric J. Brandt, Eric B. Rimm

**Affiliations:** 1 Department of Nutrition, Harvard T. H. Chan School of Public Health, Boston, Massachusetts; 2 Department of International Health, Johns Hopkins Bloomberg School of Public Health, Baltimore, Maryland; 3 Department of Health Policy and Management, Johns Hopkins Bloomberg School of Public Health, Baltimore, Maryland; 4 Division of Cardiovascular Medicine, University of Michigan, Ann Arbor; 5 Department of Epidemiology, Harvard T. H. Chan School of Public Health, Boston, Massachusetts; 6 Channing Division of Network Medicine, Department of Medicine, Brigham and Women’s Hospital and Harvard Medical School, Boston, Massachusetts

## Abstract

This cross-sectional study examines the associations among household food security, Supplemental Nutrition Assistance Program participation, and cardiovascular health among 2013-2018 National Health and Nutrition Examination Survey participants.

## Introduction

The American Heart Association recently updated its definition of ideal cardiovascular health (CVH) to encompass 8 health behaviors and clinical measures (ie, Life’s Essential 8 [LE8]) and to centralize the importance of social determinants of health in cardiovascular disease prevention.^1^ Food insecurity, a condition of limited food availability due to insufficient resources, is one critical social determinant of health affecting 10% of US households.^2^ Few studies have examined food insecurity in relation to multidimensional cardiovascular outcomes. Furthermore, this association may be complicated by participation in the Supplemental Nutrition Assistance Program (SNAP), which aims to alleviate food insecurity and also targets populations most vulnerable to poverty and poor health. This study examined the associations among household food security, SNAP participation, and LE8 as a measure of ideal CVH.

## Methods

We used data from the 2013-2018 National Health and Nutrition Examination Survey, a continuous, multistage survey representative of the noninstitutionalized US population. The study population included 11 520 nonpregnant adults 20 years and older. Analysis of publicly available secondary data was deemed exempt from approval by the Harvard University Institutional Review Board, and informed consent was not required. The study followed the STROBE reporting guideline.

The primary exposure was household food security, measured using the US Household Food Security Survey Module, and categorized as high, marginal, low, and very low using US Department of Agriculture guidelines.^2^ The secondary exposure was household SNAP participation in the last 12 months. Nonparticipants were classified as low income (≤130% of the federal poverty level) or higher income (>130% of the federal poverty level).

The primary outcomes were the LE8 measures (physical activity, diet, tobacco use, sleep health, body mass index [BMI], blood pressure, and serum glucose and lipid levels).^1^ Published algorithms were applied to create component scores ranging from 0 to 100.^3^ The overall LE8 score is the mean of all component scores.

We used multivariable linear regression models to examine differences in continuous overall and component LE8 scores by household food security and SNAP participation and multivariable logistic regression to examine joint associations between household food security and SNAP participation with moderate or ideal CVH (overall LE8 score ≥50). Models were adjusted for sociodemographic covariates. Complex sampling weights were applied to all analyses.

## Results

Among the 11 520 participants (51.7% women and 48.3% men; mean [SE] age, 47.9 [0.4] years), 9.9% had marginal food security, 10.2% had low food security, and 6.5% had very low food security. Compared with adults with high food security (66.9 [0.4]), those with marginal (65.4 [0.6]), low (63.9 [0.8]), and very low (62.3 [0.8]) food security had lower overall mean (SE) LE8 scores in a dose-response manner (*P* < .001 for trend) (Table). Greater severity of food insecurity was also inversely associated with diet, tobacco use, sleep health, BMI, and serum glucose scores (*P* < .05 for trend). SNAP participants had the lowest overall mean (SE) LE8 score (62.8 [0.6]) compared with low- (65.5 [0.7]) and higher-income (65.4 [0.5]) nonparticipants. SNAP participation was also associated with lower scores for diet, tobacco, sleep, and BMI.

**Table.** Least Square Means of Overall and Component Life’s Essential 8 Scores by Household Food Security Status and Supplemental Nutrition Assistance Program Participation^a^

Status	LE8 domain score								
	Diet	Physical activity	Tobacco use	Sleep health	BMI	Serum lipid level^b^	Serum glucose level^c^	Blood pressure^d^	Total
Food security									
High	43.0 (0.8)	57.0 (1.4)	70.9 (1.1)	81.5 (0.7)	60.5 (0.7)	65.5 (0.8)	77.6 (0.7)	80.0 (0.5)	66.9 (0.4)
Marginal	40.4 (1.2)	59.7 (1.8)	66.7 (2.1)^e^	79.5 (1.1)	57.4 (1.5)^e^	65.7 (1.4)	74.0 (1.2)	80.4 (0.7)	65.4 (0.6)^e^
Low	40.1 (2.0)	57.2 (2.2)	64.2 (2.0)^f^	78.1 (1.1)	54.6 (1.6)^e^	65.9 (1.3)	73.9 (1.2)^e^	78.5 (1.1)	63.9 (0.8)^e^
Very low	33.8 (1.5)^e^^,^^f^	58.8 (2.6)	56.0 (2.1)^e^^,^^f^	76.8 (1.6)^e^^,^^f^	58.0 (1.8)^e^	63.9 (2.0)	74.3 (1.7)^f^	77.7 (1.4)	62.3 (0.8)^e^^,^^f^
SNAP participation									
Higher-income nonparticipant	39.7 (1.1)	59.1 (1.2)	66.6 (1.3)	80.5 (0.7)	58.7 (1.3)	65.5 (1.2)	75.6 (1.0)	78.6 (0.7)	65.4 (0.5)
Low-income nonparticipant	40.8 (1.5)	57.3 (2.4)	69.2 (2.0)	78.8 (1.2)	59.3 (1.7)	64.1 (1.6)	74.5 (1.3)	80.9 (1.2)	65.5 (0.7)
SNAP participant	38.0 (1.6)^g^	56.8 (1.6)	58.5 (2.0)^g^^,^^h^	76.1 (1.0)^g^^,^^h^	54.9 (1.2)^g^^,^^h^	65.2 (1.2)	73.8 (1.2)	79.6 (0.7)	62.8 (0.6)^g^^,^^h^

Abbreviations: BMI, body mass index (calculated as weight in kilograms divided by height in meters squared); LE8, Life’s Essential 8; SNAP, Supplemental Nutrition Assistance Program.

^a^ Includes 11 520 participants from the 2013-2018 National Health and Nutrition Examination Surveys. Data are expressed as mean (SE). Means were adjusted for age, sex, race and ethnicity, educational attainment, marital status, and ratio of family income to poverty. Scores of 50 or greater indicate moderate to ideal cardiovascular health.

^b^ Calculated from non–high-density lipoprotein (HDL) cholesterol level in measured total and HDL nonfasting cholesterol levels.

^c^ Calculated from hemoglobin A_1c_ levels assayed from whole blood biospecimens.

^d^ Calculated from the mean of 3 readings (systolic and diastolic).

^e^ Significant difference from the group with high food security (*P* < .05).

^f^ Significant trend in outcome by severity of food insecurity (*P* < .05).

^g^ Significant difference between SNAP participants and low-income nonparticipants (*P* < .05).

^h^ Significant difference between SNAP participants and higher-income nonparticipants (*P* < .05).

SNAP participation modified the association between household food security and moderate to ideal CVH (Figure). Greater severity of food insecurity was associated with lower probabilities of moderate to ideal CVH for all SNAP participation groups. SNAP participants with very low food security had the lowest probability of moderate to ideal CVH (74.9% [95% CI, 66.4%-81.8%]).

**Figure.**
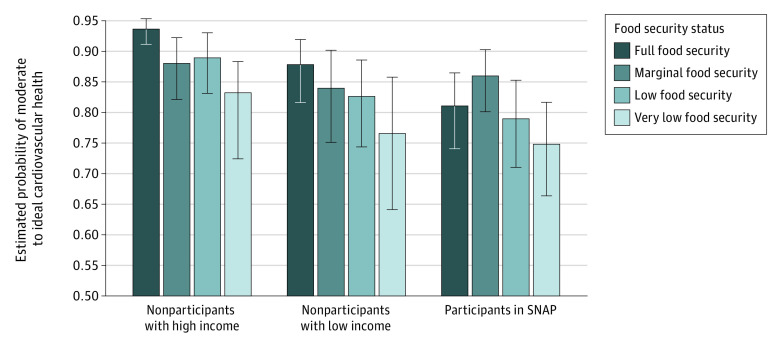
Estimated Probabilities of Moderate to Ideal Cardiovascular Health Estimates are based on Life’s Essential 8 scores of 50 or greater among 11 520 nonpregnant adults (aged ≥20 years) by household food security and Supplemental Nutrition Assistance Program (SNAP) participation, adjusted for age, sex, race and ethnicity, educational attainment, marital status, and ratio of family income to poverty. Error bars indicate 95% CIs.

## Discussion

The findings of this nationally representative cross-sectional study suggest that food insecurity and SNAP participation may be critical barriers to CVH, specifically for diet, tobacco use, sleep health, and BMI. SNAP participants with very low food security had the lowest probability of moderate to ideal CVH, suggesting nutrition-forward policies may improve food security and CVH among program participants.^4^ This study is cross-sectional, which precludes temporality or causality. Our results align with findings from prior studies, supporting the robustness of the results.^5,6^ Programmatic and policy interventions should consider more holistic approaches to improve behavioral disparities among populations at risk of food insecurity.

## Notes

Supplement.
Data Sharing Statement
