# Supplementary material for: Disparities in Cardiovascular Health by Food Security Status and Supplemental Nutrition Assistance Program Participation Using Life’s Essential 8 Metrics
Source: JAMA Netw Open. 2023 Jun 30;6(6):e2321375. doi: 10.1001/jamanetworkopen.2023.21375 (PMC10314299; doi:10.1001/jamanetworkopen.2023.21375)
Supplement: Supplement. — Data Sharing Statement [file jamanetwopen-e2321375-s001.pdf]

Data Sharing Statement

Leung. Disparities in Cardiovascular Health by Food Security Status and Supplemental Nutrition Assistance Program Participation Using Life's Essential 8 Metrics. *JAMA Netw Open*. Published June 30, 2023. doi:10.1001/jamanetworkopen.2023.21375

Data
Data available: No

Additional Information
Explanation for why data not available: Data for the study are publicly available from the National Center for Health Statistics
